# Magnetic resonance imaging of healthy and diseased brain networks

**DOI:** 10.3389/fnhum.2014.00890

**Published:** 2014-11-03

**Authors:** Yong He, Alan Evans

**Affiliations:** ^1^State Key Laboratory of Cognitive Neuroscience and Learning, IDG/McGovern Institute for Brain Research, Beijing Normal UniversityBeijing, China; ^2^Center for Collaboration and Innovation in Brain and Learning Sciences, Beijing Normal UniversityBeijing, China; ^3^McConnell Brain Imaging Centre, Montreal Neurological Institute, McGill University MontrealMontreal, QC, Canada

**Keywords:** connectomics, connectivity, graph theory, small-world, MRI

An important aspect of neuroscience is to characterize the underlying connectivity patterns of the human brain. Recent advances in magnetic resonance imaging (MRI) techniques (e.g., structural MRI, diffusion MRI, and functional MRI) and network analysis approaches such as graph theory have allowed to investigate the patterns of structural and functional connectivity of human brain (i.e., human connectome, Sporns et al., 2005). Using imaging connectomics, many studies have demonstrated that large-scale human brain networks have many non-trivial topological properties such as small-worldness, modular structure, and highly connected hubs. Moreover, these quantifiable network properties significantly correlate with behavioral, environmental, and genetic factors, and change with age, learning, and disease. Network analysis of neuroimaging data is opening up a new avenue of research into the understanding of the organizational principles of the brain that will be crucial for all basic scientists and clinical researchers. Such approaches are fascinating but there are a number of challenging issues in the extraction of reliable brain networks from various imaging modalities and the analyses of the topological properties, such as the definitions of network nodes and edges, and the reproducibility of the network analysis results. Nonetheless, the field of imaging connectomics has significantly advanced in the past several years, largely due to the rapid development of network models, tools and methodologies, and their widespread applications in cognitive and clinical research.

In this research topic, we aimed at compiling works representing the state of the art in structural and functional brain networks in healthy and disease populations using neuroimaging data. We collected 29 articles that were from a number of internationally recognized scientists who have made significant contributions to this field. The article types are diverse and the topics covered various research directions including imaging techniques, computational modeling, network analysis approaches and tools, and applications.

- We assembled one hypothesis and theory article, one perspective article, and four review articles. In the hypothesis and theory article, Horwitz et al. (2013) highlighted recent efforts toward using large-scale neural modeling to explore the relationship between structural connectivity and functional/effective connectivity. Structural connectivity and functional/effective connectivity are the most fundamental concepts for the descriptions of brain networks, but their relationship is elusive. Horwitz et al. (2013) emphasized that the alteration of structural connectivity known in models does not necessarily result in matching changes in functional/effective connectivity and vice versa, suggesting that caution should be taken in the result interpretation of structural-functional connectivity relationship. Upon summarizing three commonly used strategies of imaging connectomics as biomarkers of brain diseases, Kaiser (2013) presented a novel fourth option for future disease biomarker studies, i.e., dynamic connectomes that use computational models of simulated brain activity based on structural connectivity rather than the structural connectome itself. Among the four review articles, Li et al. (2014) summarized brain connectivity studies related to the default-mode network (DMN) in the fields of social understanding: emotion perception, empathy, theory of mind, and morality, and suggested the vital roles of the DMN in this domain; Hoff et al. (2014) reviewed resting fMRI studies representing developmental changes in the functional brain networks from 20 weeks of gestation onwards, highlighting different developmental rates of network connectivity in different brain systems; De La Fuente et al. (2013) provided a systematic review regarding brain connectivity studies in attention-deficit/hyperactivity disorder and proposed that the roles of the subcortical structures and their structural/functional pathways in this developmental disorder should be further studied; Bernhardt et al. (2013) reviewed the application of multi-modal imaging techniques and brain connectivity approaches in temporal lobe epilepsy, specifically highlighting findings from graph-theoretical analysis that assessed the topological organization of brain networks. Together, these articles suggest that the combination of multi-modal imaging techniques and advanced network analysis approaches such as computational modeling and graph theory provides unique opportunities to enrich our understanding of biological mechanisms in healthy and diseased brains. In these articles, a number of important research directions were proposed for future brain network studies based on neuroimaging data.
- We assembled 23 original research articles, which can be roughly classified into imaging and analysis methodologies, tools, and applications in various domains.

## Methodologies and tools

One compelling imaging technique study done by Mandl et al. (2013) showed that functional diffusion tensor imaging (fDTI) method is capable to robustly detect neuronal activity of human brain networks within a practically feasible time period. Using an interesting meta-analytic clustering (MaC) approach, Torta et al. (2013) found that the cingulate cortex, an important brain hub, can be parcellated into three clusters, and that the clustering pattern of this region changes across different levels of task complexity. Importantly, two resting fMRI studies by Hayasaka (2013) and Yan et al. (2013) performed comprehensive validation analyses on the effects of global signal and head motion on brain network analysis and highlighted remarkable differences between the networks with or without these corrections. Another resting fMRI study by Kollndorfer et al. (2013) showed the reproducibility of functional connectivity patterns in four frequently used scanning conditions (i.e., fixation of a black crosshair on a white screen; fixation of the center of a black screen; eyes-closed and fixation of the words “relax”), suggesting that intrinsic brain connectivity measurements are reliable across these conditions and confirmed its potential in assessing brain networks in clinical settings. Lastly, Cui et al. (2013) developed a novel MATLAB toolbox named “Pipeline for Analyzing braiN Diffusion imAges” (PANDA) for fully automated processing of diffusion MRI data of the human brain, which substantially simplifies the image processing and facilitates imaging connectome studies.

## Task modulation and individual differences

Three papers utilized network analysis to study task-related modulation and individual differences. An important issue in connectomics is to understand how brain networks measured during resting state are reorganized by various task performances. Di et al. (2013) addressed this issue by studying meta-analytic coactivation patterns among regions based upon published neuroimaging studies and compared with those derived from resting fMRI data. They observed that the coactivation network showed greater global efficiency, smaller clustering coefficient, and lower modularity than the resting-state network, indicating a more efficient global information transmission during task performing. These findings highlighted topological reconfiguration of large-scale brain networks between task and resting-state conditions. Using cortical thickness covariance analysis of structural MRI data, Krishnadas et al. (2013) examined the association between neighborhood level deprivation and brain network structure, and found that the most deprived group showed modular patterns different from the least deprived group. These results provide preliminary evidence that the structural networks of the human brain might be associated with socioeconomic deprivation. Another interesting fMRI study done by Gao et al. (2013) demonstrated the association between the topological organization of whole-brain functional networks and individual differences such as extraversion and neuroticism.

## Normal development and aging

Four papers directly examined age-related changes in structural brain networks. Using DTI, Mishra et al. (2013) investigated the relationship between 10 major white matter tracts with distinctive functions in neonates and children around puberty: Stronger microstructural inter-tract correlations were observed during development from birth to puberty, indicating heterogeneous but organized myelination processes. Using DTI data of a large sample (*n* = 180), Chen et al. (2013b) specifically examined the topological organization of white matter networks in typically-developing participants, including early childhood (6.0–9.7 years), late childhood (9.8–12.7 years), adolescence (12.9–17.5 years), young adult (17.6–21.8 years), and adult (21.9–29.6 years). They showed that most prominent changes in the topological efficiencies of developing brain networks occur at late childhood and adolescence. Using structural MRI, Li et al. (2013) exclusively examined eight structural covariance networks in 240 healthy participants aged 18–89 years, and charted the age-related network reorganization involving language-related speech and semantics processing, executive control network (ECN) and the DMN network. In a longitudinal structural MRI study, Wu et al. (2013) illustrated age-related dynamic changes in network connectivity: The structural covariance networks develop into a fast distribution from young to middle age (~50 years old) and eventually become a fast localization in the old age. These studies significantly increased our understanding of structural substrates underlying normal development and aging.

## Brain disorders

Various kinds of brain disorders were investigated using network analysis approaches. Using combined resting fMRI and structural MRI, Chen et al. (2013a) demonstrated that the insular module in the cognitively normal group broken down to pieces in patients with Alzheimer's disease and that the corresponding gray matter concentration was significantly lower in the patient group. Importantly, they proposed a quantitative index by integrating the functional connectivity changes and structural changes in this brain module, which shows potential as diagnostic biomarkers of Alzheimer's disease at the single-subject level. Using an independent component analysis (ICA) and a dual regression technique, Rytty et al. (2013) found that patients with behavioral variant of frontotemporal dementia showed abnormally increased resting fMRI connectivity in the dorsal attention network and DMN network, which might provide neuronal basis for impairments in executive functions and attention in patients.

Two fMRI studies directly examined schizophrenic brain networks. Using a monetary incentive delay experimental task, White et al. (2013) observed dysregulated but not decreased functional activities in the salience network (SN) in schizophrenia, which offers physiological explanations for the delusional thought formation in this disease. Using resting fMRI data, Anderson and Cohen (2013) showed that patients with schizophrenia had disrupted functional network topology as characterized by less clustering and lower small-world connectivity. Specifically, a support vector machine classifier based on these connectivity features could discriminate individuals with schizophrenia patients from healthy controls with 65% accuracy. Worth noting is that Ottet et al. (2013) investigated topological patterns of DTI-based structural brain networks in the 22q11.2 deletion syndrome (22q11.2DS), usually considered to be a homogeneous genetic sub-type of schizophrenia. They showed a loss of global degree connectivity in brain hubs of the patients (~58%) and the association between local efficiency of several key regions (the Broca's area, the Wernicke area and the dorsolateral prefrontal cortex) and symptom severity. These results provide evidence for the targeted alterations of specific brain hubs associated with language and thought regulation in individuals with a genetic risk for schizophrenia, which may help to understand the biological mechanism underlying hallucination.

Three studies highlighted brain network dysfunctions in mood disorders. Using resting fMRI data and high-model order ICA, Doll et al. (2013) examined the interactions across three major intrinsic networks of the human brain (i.e., SN, DMN, and ECN) in borderline personality disorder. They observed disrupted intra-network connectivity in all three networks and a strong shift of inter-network connectivity from networks involved in cognitive control to those in motion processing, potentially reflecting the persistent instability of emotion regulation in patients. Also using the ICA approach, Manoliu et al. (2014) reported decreased intra-network connectivity within the SN in patients with major depressive disorder and that the extent of decrease was associated with severity of symptoms. Moreover, inter-network connections were decreased between the DMN and ECN, and were increased between the SN and DMN. These findings suggest an important link between aberrant salience mapping and network coordination involving cognitive processes and psychopathology in depression. Using graph theoretical analysis based on cortical thickness from structural MRI, Kim et al. (2013) provided direct evidence for disparity between dorsal and ventral networks in cortico-striato-thalamic circuit in patients with obsessive-compulsive disorder.

Network abnormalities were also demonstrated in other brain disorders including traumatic brain injury and pathological gambling. Combining task-related fMRI functional connectivity with DTI structural connectivity, Caeyenberghs et al. (2013) for the first time examined topological correlations of structural and functional brain networks in patients with traumatic brain injury and healthy controls. They found that graph metrics and hubs of brain networks showed no agreement in both groups, suggesting that the topological properties of functional networks could not be solely accounted for by the structural networks. However, prediction accuracy in switching performance could be improved by combining brain connectivity information from both imaging modalities. Using graph-based network analysis of resting fMRI, Tschernegg et al. (2013) reported that at the nodal level, pathological gambler had reduced clustering coefficient and local efficiency in the left paracingulate cortex and the left supplementary motor area, but an increased node betweenness for these regions, suggesting that regions in the reward system show reduced functional segregation but enhanced functional integration. These findings provide direct evidence for the topological abnormalities of the brain networks associated with pathological gambling.

Overall, the wealth of methods and applications covered by this research topic shows the exciting recent advances of multi-modal neuroimaging and network analysis as powerful approaches to study the neuronal circuits of healthy and diseased populations. We anticipate that these works will provide critical insights into the field of imaging brain networks. Lastly, we would like to thank all of the authors, reviewers and the Frontiers editorial office for their important contributions to this Research Topic.

### Conflict of interest statement

The authors declare that the research was conducted in the absence of any commercial or financial relationships that could be construed as a potential conflict of interest.

## References

[B1] Anderson A. Cohen M. S. (2013). Decreased small-world functional network connectivity and clustering across resting state networks in schizophrenia: an fMRI classification tutorial. Front. Hum. Neurosci. 7:520. 10.3389/fnhum.2013.00520 24032010 PMC3759000

[B2] Bernhardt B. C. Hong S. Bernasconi A. Bernasconi N. (2013). Imaging structural and functional brain networks in temporal lobe epilepsy. Front. Hum. Neurosci. 7:624. 10.3389/fnhum.2013.00624 24098281 PMC3787804

[B3] Caeyenberghs K. Leemans A. Leunissen I. Michiels K. Swinnen S. P. (2013). Topological correlations of structural and functional networks in patients with traumatic brain injury. Front. Hum. Neurosci. 7:726. 10.3389/fnhum.2013.00726 24204337 PMC3817367

[B4] Chen G. Zhang H. Y. Xie C. Chen G. Zhang Z. J. Li S. J.. (2013a). Modular reorganization of brain resting state networks and its independent validation in Alzheimer's disease patients. Front. Hum. Neurosci. 7:456. 10.3389/fnhum.2013.00456 23950743 PMC3739061

[B5] Chen Z. Liu M. Gross D. W. Beaulieu C. (2013b). Graph theoretical analysis of developmental patterns of the white matter network. Front. Hum. Neurosci. 7:716. 10.3389/fnhum.2013.00716 24198774 PMC3814848

[B6] Cui Z. Zhong S. Xu P. He Y. Gong G. (2013). PANDA: a pipeline toolbox for analyzing brain diffusion images. Front. Hum. Neurosci. 7:42. 10.3389/fnhum.2013.00042 23439846 PMC3578208

[B7] De La Fuente A. Xia S. Branch C. Li X. (2013). A review of attention-deficit/hyperactivity disorder from the perspective of brain networks. Front. Hum. Neurosci. 7:192. 10.3389/fnhum.2013.00192 23720619 PMC3654209

[B8] Di X. Gohel S. Kim E. H. Biswal B. B. (2013). Task vs. rest-different network configurations between the coactivation and the resting-state brain networks. Front. Hum. Neurosci. 7:493. 10.3389/fnhum.2013.00493 24062654 PMC3775427

[B9] Doll A. Sorg C. Manoliu A. Woller A. Meng C. Forstl H.. (2013). Shifted intrinsic connectivity of central executive and salience network in borderline personality disorder. Front. Hum. Neurosci. 7:727. 10.3389/fnhum.2013.00727 24198777 PMC3812906

[B10] Gao Q. Xu Q. Duan X. Liao W. Ding J. Zhang Z.. (2013). Extraversion and neuroticism relate to topological properties of resting-state brain networks. Front. Hum. Neurosci. 7:257. 10.3389/fnhum.2013.00257 23781183 PMC3678091

[B11] Hayasaka S. (2013). Functional connectivity networks with and without global signal correction. Front. Hum. Neurosci. 7:880. 10.3389/fnhum.2013.00880 24385961 PMC3866385

[B12] Hoff G. E. Van den Heuvel M. P. Benders M. J. Kersbergen K. J. De Vries L. S. (2014). On development of functional brain connectivity in the young brain. Front. Hum. Neurosci. 7:650. 10.3389/fnhum.2013.00650 24115929 PMC3792361

[B13] Horwitz B. Hwang C. Alstott J. (2013). Interpreting the effects of altered brain anatomical connectivity on fMRI functional connectivity: a role for computational neural modeling. Front. Hum. Neurosci. 7:649. 10.3389/fnhum.2013.00649 24273500 PMC3822330

[B14] Kaiser M. (2013). The potential of the human connectome as a biomarker of brain disease. Front. Hum. Neurosci. 7:484. 10.3389/fnhum.2013.00484 23966935 PMC3744009

[B15] Kim S. G. Jung W. H. Kim S. N. Jang J. H. Kwon J. S. (2013). Disparity between dorsal and ventral networks in patients with obsessive-compulsive disorder: evidence revealed by graph theoretical analysis based on cortical thickness from MRI. Front. Hum. Neurosci. 7:302. 10.3389/fnhum.2013.00302 PMC3699763 23840184

[B16] Kollndorfer K. Fischmeister F. P. Kasprian G. Prayer D. Schopf V. (2013). A systematic investigation of the invariance of resting-state network patterns: is resting-state fMRI ready for pre-surgical planning? Front. Hum. Neurosci. 7:95. 10.3389/fnhum.2013.00095 23532457 PMC3607808

[B17] Krishnadas R. Kim J. McLean J. Batty G. D. McLean J. S. Millar K.. (2013). The envirome and the connectome: exploring the structural noise in the human brain associated with socioeconomic deprivation. Front. Hum. Neurosci. 7:722. 10.3389/fnhum.2013.00722 24273501 PMC3824100

[B18] Li W. Mai X. Liu C. (2014). The default mode network and social understanding of others: what do brain connectivity studies tell us. Front. Hum. Neurosci. 8:74. 10.3389/fnhum.2014.00074 24605094 PMC3932552

[B19] Li X. Pu F. Fan Y. Niu H. Li S. Li D. (2013). Age-related changes in brain structural covariance networks. Front. Hum. Neurosci. 7:98. 10.3389/fnhum.2013.00098 23532684 PMC3607831

[B20] Mandl R. C. Schnack H. G. Zwiers M. P. Kahn R. S. Hulshoff Pol H. E. (2013). Functional diffusion tensor imaging at 3 Tesla. Front. Hum. Neurosci. 7:817. 10.3389/fnhum.2013.00817 24409133 PMC3847896

[B21] Manoliu A. Meng C. Brandl F. Doll A. Tahmasian M. Scherr M.. (2014). Insular dysfunction within the salience network is associated with severity of symptoms and aberrant inter-network connectivity in major depressive disorder. Front. Hum. Neurosci. 7:930. 10.3389/fnhum.2013.00930 PMC3896989 24478665

[B22] Mishra V. Cheng H. Gong G. He Y. Dong Q. Huang H. (2013). Differences of inter-tract correlations between neonates and children around puberty: a study based on microstructural measurements with DTI. Front. Hum. Neurosci. 7:721. 10.3389/fnhum.2013.00721 PMC3810597 24194711

[B23] Ottet M. C. Schaer M. Debbane M. Cammoun L. Thiran J. P. Eliez S. (2013). Graph theory reveals dysconnected hubs in 22q11DS and altered nodal efficiency in patients with hallucinations. Front. Hum. Neurosci. 7:402. 10.3389/fnhum.2013.00402 24046733 PMC3763187

[B24] Rytty R. Nikkinen J. Paavola L. Abou Elseoud A. Moilanen V. Visuri A.. (2013). GroupICA dual regression analysis of resting state networks in a behavioral variant of frontotemporal dementia. Front. Hum. Neurosci. 7:461. 10.3389/fnhum.2013.00461 23986673 PMC3752460

[B25] Sporns O. Tononi G. Kötter R. (2005). The human connectome: a structural description of the human brain. PLoS Comput. Biol. 1:e42. 10.1371/journal.pcbi.0010042 16201007 PMC1239902

[B26] Torta D. M. Costa T. Duca S. Fox P. T. Cauda F. (2013). Parcellation of the cingulate cortex at rest and during tasks: a meta-analytic clustering and experimental study. Front. Hum. Neurosci. 7:275. 10.3389/fnhum.2013.00275 23785324 PMC3682391

[B27] Tschernegg M. Crone J. S. Eigenberger T. Schwartenbeck P. Fauth-Buhler M. Lemenager T.. (2013). Abnormalities of functional brain networks in pathological gambling: a graph-theoretical approach. Front. Hum. Neurosci. 7:625. 10.3389/fnhum.2013.00625 24098282 PMC3784685

[B28] White T. P. Gilleen J. Shergill S. S. (2013). Dysregulated but not decreased salience network activity in schizophrenia. Front. Hum. Neurosci. 7:65. 10.3389/fnhum.2013.00065 23471456 PMC3590462

[B29] Wu K. Taki Y. Sato K. Qi H. Kawashima R. Fukuda H. (2013). A longitudinal study of structural brain network changes with normal aging. Front. Hum. Neurosci. 7:113. 10.3389/fnhum.2013.00113 23565087 PMC3615182

[B30] Yan C. G. Craddock R. C. He Y. Milham M. P. (2013). Addressing head motion dependencies for small-world topologies in functional connectomics. Front. Hum. Neurosci. 7:910. 10.3389/fnhum.2013.00910 24421764 PMC3872728

